# Preclinical Evaluation of Dual-Fiber Laser Ablation for Controlled Thermal Necrosis in Bovine Prostate Tissue Using the EchoLaser X4 System

**DOI:** 10.7759/cureus.106904

**Published:** 2026-04-12

**Authors:** Rafael Aldaya Bourricaudy, Juan Varela, Victoria Bird

**Affiliations:** 1 Urology, University of Florida College of Medicine, Gainesville, USA; 2 Urology, National Medical Association and Research Group, Gainesville, USA; 3 Urology, HCA Florida North Florida Hospital, Gainesville, USA; 4 Urology, Urologic Integrated Care, Gainesville, USA

**Keywords:** benign prostatic hyperplasia, dual-fiber laser, echolaser x4, focal laser ablation, minimally invasive urology, prostate tissue, thermal ablation

## Abstract

Introduction

Benign prostatic hyperplasia is a common condition among aging men and a major cause of lower urinary tract symptoms that can significantly impact quality of life. The condition often leads to increased healthcare utilization and may require intervention when medical therapy is insufficient. Focal laser ablation has emerged as a minimally invasive technique that enables targeted tissue destruction while preserving surrounding structures. The EchoLaser X4 system (Elesta S.p.A., Calenzano, Florence, Italy) utilizes a dual-fiber configuration designed to optimize thermal distribution and lesion symmetry.

Methods

Seven bovine prostate specimens underwent ex vivo laser ablation using the EchoLaser X4 system. Two optical fibers were positioned 10 mm apart and inserted 2 cm into the tissue. Each fiber delivered up to 1000 J at 5 W. Temperature measurements were recorded at 300, 500, 800, and 1000 J. Lesion dimensions were measured in three axes, and volumes were calculated using ellipsoid geometry.

Results

Temperature increased in a predictable manner with escalating energy delivery, reaching mean values of 29.6°C, 37.3°C, 47.5°C, and 56.4°C at 300, 500, 800, and 1000 J, respectively. The mean necrotic volume was 2,457 mm³ (±184), with lesions demonstrating consistent ellipsoidal morphology. No evidence of thermal runaway, vaporization, or carbonization was observed.

Conclusion

Dual-fiber laser ablation with the EchoLaser X4 system produced controlled, reproducible tissue necrosis, supporting further investigation as a focal therapy modality for benign prostatic hyperplasia.

## Introduction

Benign prostatic hyperplasia (BPH) demonstrates a strong age-dependent prevalence and represents a significant health burden in the United States, with approximately 25% of men affected overall, with prevalence increasing from 5-6% in men aged 40-64 years to 29-33% in men aged 65 years or older [1,2]. Histologic prevalence rises further with age, reaching up to 90% in men over 80 years, although not all individuals develop clinically significant symptoms [2,3]. As the disease progresses, many patients require intervention when medical therapy becomes insufficient. Clinically, BPH is a major contributor to lower urinary tract symptoms, including urinary frequency, urgency, nocturia, and incomplete bladder emptying, which can significantly impair quality of life and sleep. The condition is also associated with increased healthcare utilization, including frequent outpatient visits, long-term pharmacologic therapy, and surgical intervention in advanced cases [1]. In addition to symptom burden, progressive prostatic enlargement may contribute to bladder outlet obstruction, urinary retention, and recurrent urinary tract infections, further emphasizing the need for effective and durable treatment strategies [1-3].

Traditional surgical approaches, while effective, may be associated with morbidity and unintended effects on surrounding anatomical structures. As a result, minimally invasive therapies have gained increasing attention, particularly those that allow for targeted treatment while preserving normal tissue [1]. These approaches aim to reduce perioperative morbidity, shorten recovery time, and maintain sexual and urinary function while achieving adequate relief of obstruction. Focal laser ablation has emerged as a promising modality due to its ability to deliver controlled thermal energy to specific regions of tissue. This approach enables precise tissue destruction while minimizing collateral damage and has been successfully applied across multiple organ systems [4-6]. The ability to create localized zones of coagulative necrosis while preserving surrounding tissue makes laser-based therapies particularly attractive for focal prostate treatment.

Recent studies have demonstrated the feasibility and safety of transperineal laser ablation (TPLA) for BPH, with favorable outcomes and low complication rates [7-10]. These investigations have reported predictable ablation zones, improvement in urinary symptoms, and minimal perioperative morbidity. Additional investigations have further supported its clinical applicability and effectiveness in achieving controlled tissue necrosis. The use of multiple fibers has been shown to improve energy distribution and lesion geometry, enhancing both treatment efficiency and reproducibility [11-15]. Multi-fiber configurations allow for overlapping thermal fields, which may reduce temperature gradients and promote uniform tissue destruction. Dual-fiber configurations in particular allow for more uniform thermal fields and predictable ablation zones [11,13].

The EchoLaser X4 system (Elesta S.p.A., Calenzano, Florence, Italy) incorporates a dual-fiber design intended to optimize thermal distribution and improve lesion symmetry. However, further evaluation in structurally relevant tissue models is required. The present study aims to evaluate the thermal dynamics, lesion reproducibility, and spatial containment of dual-fiber laser ablation in bovine prostate tissue as a preclinical ex vivo model, with emphasis on controlled thermal coagulation and denaturation rather than biological necrosis. The primary objective was to characterize thermal dynamics by measuring intraparenchymal temperature changes at incremental energy levels. Secondary objectives included assessment of lesion reproducibility through calculated coagulation volumes and evaluation of spatial containment based on gross lesion geometry following dual-fiber energy delivery.

## Materials and methods

Seven bovine prostate specimens were obtained post-mortem and used for the ex vivo laser ablation experiments. This study was designed as a preclinical pilot feasibility experiment. Bovine prostate tissue was selected for its structural similarity and sufficient volume to evaluate thermal distribution and lesion geometry in preclinical ablation studies. A formal sample size calculation was not performed, given the exploratory nature of the study. Specimens were selected using convenience sampling based on availability. Inclusion criteria consisted of intact bovine prostate tissue with preserved architecture and sufficient volume to accommodate dual-fiber placement. Specimens with structural disruption, prior incision, fragmentation, or inadequate size for standardized fiber spacing were excluded. All tissue samples were handled in accordance with institutional standards for non-living biological materials. All ex vivo experiments were conducted at the Urologic Integrated Care, Gainesville, Florida, United States.

**Figure 1:**
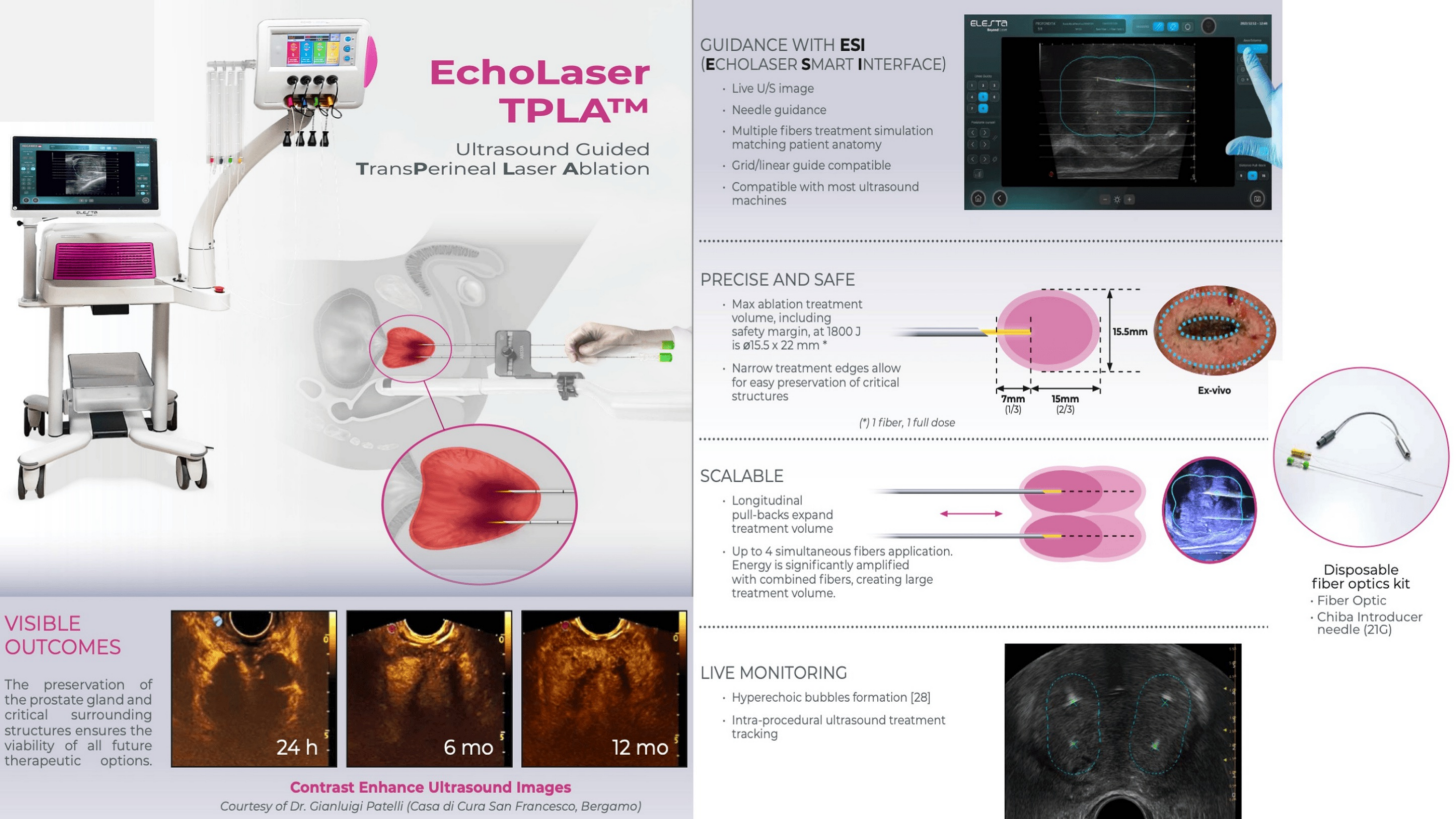
EchoLaser X4 system and dual-fiber transperineal laser ablation configuration Representative images of the EchoLaser X4 system and transperineal laser ablation workflow demonstrating system components, fiber optics, and dual-fiber placement strategy as applied in clinical (human) use. The system enables parallel fiber insertion with controlled spacing and depth to generate overlapping ellipsoidal ablation zones under image guidance. Image credit: Reproduced with permission from Rocamed SAM, Monaco.

Focal laser ablation was performed using the EchoLaser X4 system (Elesta S.p.A., Calenzano, Florence, Italy; Figure 1).

Two optical fibers were inserted into each specimen to a depth of 2 cm and positioned 10 mm apart. The selected spacing and insertion depth were based on prior experimental and clinical investigations, demonstrating that multi-fiber configurations with approximately 8-12 mm spacing produce uniform thermal overlap and reproducible ellipsoidal lesion geometry while minimizing excessive heat diffusion [11-13]. The 2 cm insertion depth was selected to allow adequate intraparenchymal placement and to replicate previously described interstitial laser ablation techniques in prostate tissue models [7-10,15].

Each fiber delivered up to 1000 J at a constant power of 5 W. Temperature measurements were recorded at predefined energy levels of 300 J, 500 J, 800 J, and 1000 J. Temperature probes were positioned in a standardized configuration relative to the laser fibers and maintained at a consistent angle (approximately 60-65°) and distance from the target tissue across all specimens to ensure reproducible assessment of thermal gradients. Following ablation, specimens were sectioned along the plane of fiber insertion. thermal coagulation and denaturation regions were measured in three orthogonal dimensions, and lesion volumes were calculated using ellipsoid geometry according to the formula V = (π/6) × length × width × height. Measurements were obtained in three orthogonal planes, corresponding to the longitudinal, transverse, and vertical axes of the ablation zone. Dimensions were obtained using direct gross measurements of the sectioned specimens to ensure consistent acquisition of length, width, and height across all samples.

Tissue samples were collected from four predefined regions: entry (1.5 cm), proximal one (2 cm), proximal two (3.5 cm), and distal one (4.5 cm), and preserved for histopathological evaluation. All procedures were standardized to ensure consistent fiber placement, spacing, and energy delivery. Maximum temperatures were maintained below 100°C to prevent carbonization and tissue vaporization. Descriptive statistical analysis was performed using Microsoft Excel (Microsoft Corporation, Redmond, WA, USA). No clinical scoring systems, questionnaires, or validated assessment scales were utilized, as this was an ex vivo experimental study. 

## Results

**Figure 2:**
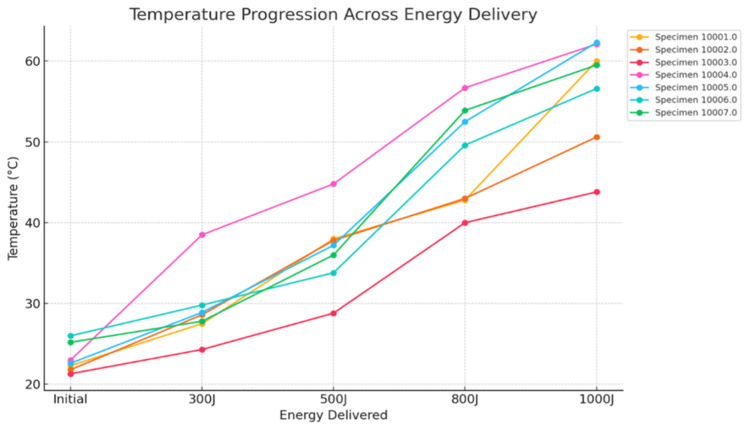
Temperature response during dual-fiber laser ablation across incremental energy delivery Temperature measurements across seven bovine prostate specimens demonstrate a consistent increase with escalating energy input from baseline to 1000 J. While individual specimens exhibit minor variability at intermediate energy levels, temperature trajectories converge at higher energy delivery, indicating reproducible thermal behavior and consistent energy deposition. No abrupt deviations or evidence of uncontrolled thermal escalation were observed across the measured range.

A progressive increase in tissue temperature was observed across all specimens as energy delivery increased from baseline to 1000 J (Figure 2, Table 1).

**Table 1:** Temperature progression and necrotic volume measurements across the seven bovine prostate specimens Temperature values represent intraparenchymal measurements recorded at baseline and incremental energy delivery levels (300 J, 500 J, 800 J, and 1000 J) during dual-fiber laser ablation. Necrotic volumes were calculated using ellipsoid geometry (V = π/6 × length × width × height) based on three orthogonal measurements of the ablation zone. Data demonstrate consistent thermal escalation and reproducible lesion formation across specimens.

Specimen	Baseline (°C)	300 J (°C)	500 J (°C)	800 J (°C)	1000 J (°C)	Necrotic Volume (mm³)
10001	22.3	27.6	37.8	43.2	59.8	2410
10002	22.1	28.1	37.5	42.9	50.5	2265
10003	21.5	24.3	28.8	39.8	43.7	2184
10004	23.2	38.5	44.7	56.8	62.4	2710
10005	22.8	29.1	36.9	52.6	62.1	2660
10006	26	29.7	33.8	49.5	56.7	2385
10007	25.3	27.9	35.9	53.9	59.2	2565

Mean temperatures were 29.6°C at 300 J, 37.3°C at 500 J, 47.5°C at 800 J, and 56.4°C at 1000 J. All specimens demonstrated an upward thermal trajectory throughout the ablation sequence. Although minor inter-specimen variability was present at intermediate energy levels, the overall pattern remained consistent, with convergence of temperature values at higher energy delivery.

The rate of temperature rise was modest between baseline and 300 J and became more pronounced between 500 J and 800 J. By 1000 J, temperatures remained below the predefined 100°C threshold in all specimens, and no abrupt surges suggestive of thermal instability were observed. These findings indicate stable heat generation throughout the ablation process.

**Figure 3:**
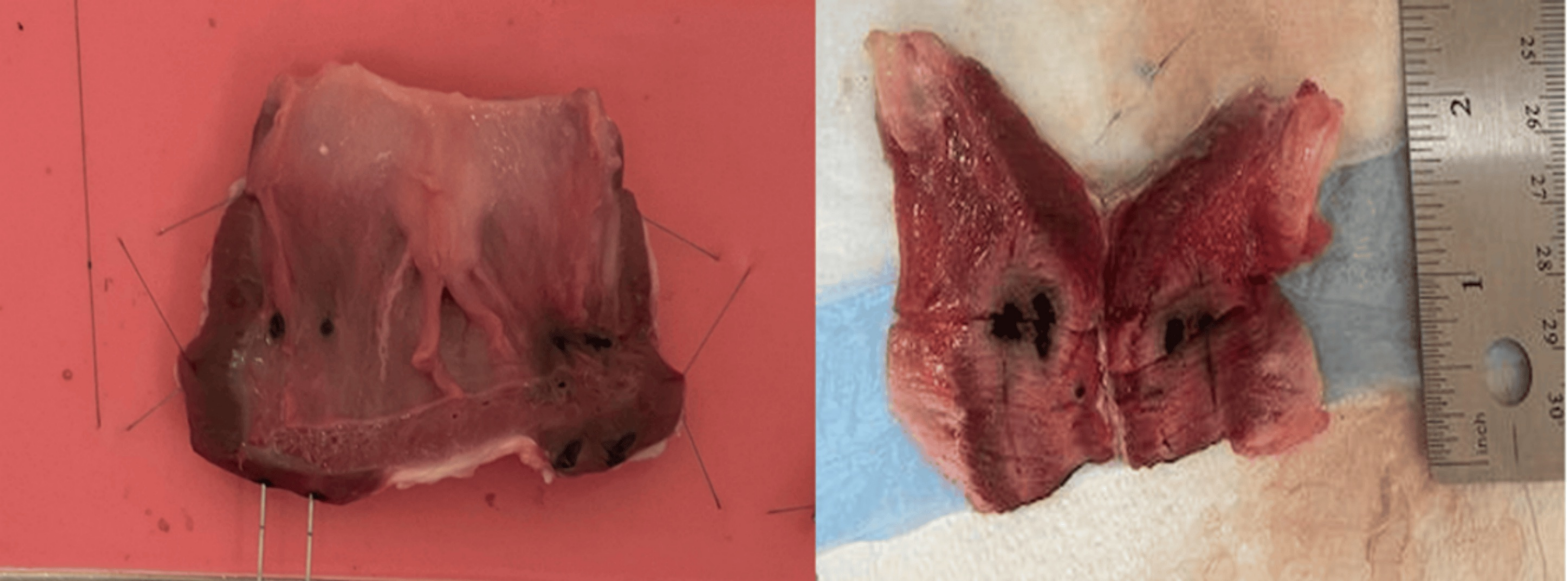
Gross tissue morphology following dual-fiber laser ablation demonstrates reproducible lesion architecture Post-ablation transverse sections of bovine prostate tissue following 1000 J energy delivery reveal well-defined zones of coagulative necrosis with consistent ellipsoidal geometry. The lesions exhibit symmetric distribution corresponding to fiber positioning, with clear demarcation between ablated and surrounding viable tissue. The extent and uniformity of tissue changes reflect controlled thermal spread and localized energy deposition.

Gross examination after sectioning demonstrated reproducible lesion formation in all ablated specimens (Figure 3).

The mean necrotic volume was 2,457 mm³ (±184), with limited variation among samples. Lesions consistently exhibited an ellipsoidal configuration, with longitudinal extension corresponding to the axis of fiber placement. The resulting ablation zones were visually symmetric and centered between the two fiber tracts.

The ablated regions showed well-demarcated zones of coagulative necrosis with homogeneous central discoloration and clearly preserved surrounding tissue architecture. Boundaries between treated and untreated tissue were distinct across all specimens. No irregular lesion contours, asymmetric extension, or discontinuous areas of injury were identified. Tissue distortion was minimal, and lesion morphology remained grossly uniform across the study cohort.

**Figure 4:**
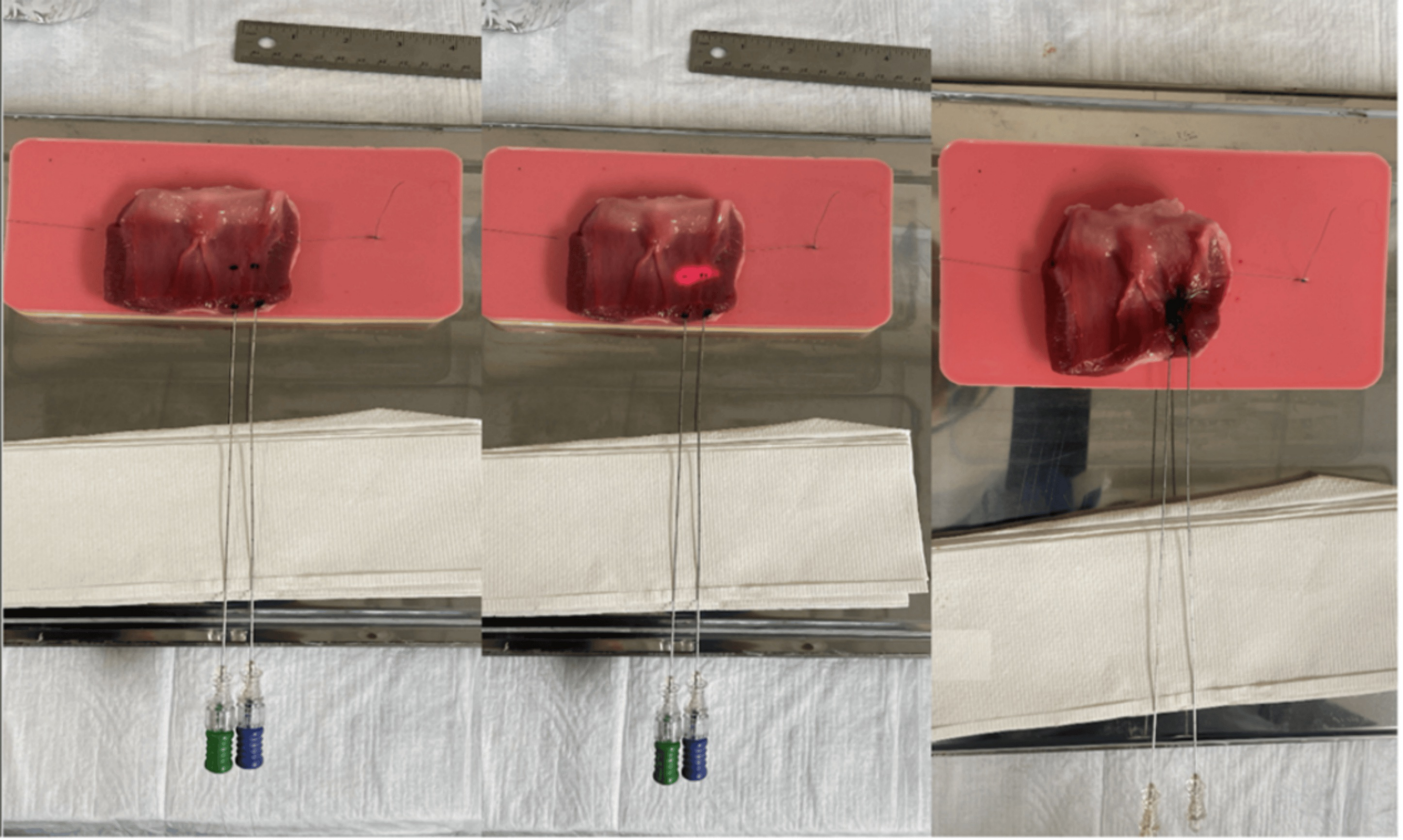
Dual-fiber configuration and sequential tissue response during laser ablation Parallel-fiber placement within the bovine prostate tissue demonstrates the spatial relationship between probe positioning and the resulting ablation pattern. Progressive changes in tissue appearance with increasing energy delivery illustrate the transition from baseline architecture to localized thermal injury, with lesion development occurring in a controlled and spatially confined manner.

Sequential gross assessment during the procedure demonstrated progressive tissue change with increasing energy exposure (Figure 4).

Baseline tissue appearance was preserved prior to activation. At intermediate energy delivery, localized discoloration became apparent around the treatment zone, corresponding to the early thermal effect. Following completion of ablation, the treated region demonstrated a visibly consolidated zone of tissue injury localized to the area between and immediately surrounding the fiber tracts.

No gross evidence of excessive carbonization or thermal runaway was observed in any specimen post-ablation. A thin rim of superficial charring adjacent to the fiber tract is an expected finding during interstitial laser ablation and was observed in some specimens. This limited surface darkening represented localized thermal coagulation at the fiber-tissue interface, rather than uncontrolled carbonization extending beyond the ablation margin; as the ex vivo non-perfused tissue model lacks blood flow and physiologic heat dissipation, which may accentuate superficial discoloration compared with in vivo conditions. No evidence of unintended extension of thermal injury beyond the intended treatment field was observed. Overall, the findings demonstrated reproducible thermal progression, consistent lesion geometry, and localized tissue effect across all seven specimens.

## Discussion

Our preclinical study demonstrates that dual-fiber laser ablation produces predictable thermal profiles and reproducible lesion geometry in bovine prostate tissue under controlled conditions. The observed consistency in both temperature progression and lesion morphology across specimens supports the reliability of this approach as a controlled thermal ablation strategy. Reproducibility in thermal delivery is a critical requirement for focal therapies, where precise energy deposition directly influences treatment efficacy and safety.

The progressive increase in temperature with escalating energy delivery observed in this study reflects the fundamental relationship between energy deposition and tissue heating in laser-based therapies. This predictable thermal response is critical for achieving controlled ablation while avoiding excessive or unintended tissue damage. Prior experimental studies evaluating interstitial laser ablation systems have similarly demonstrated that coordinated energy delivery yields stable, reproducible thermal distributions, particularly when multiple fibers are used [11-13]. Multi-fiber systems enable more uniform heat dispersion and reduce the risk of focal overheating or undertreatment. The absence of abrupt temperature fluctuations in the present study further supports the system's stability under the tested conditions and suggests controlled thermal propagation within the tissue.

The consistent ellipsoidal morphology and symmetry of the ablation zones observed across all specimens are notable findings. Lesion geometry is a key determinant of treatment efficacy in focal therapies, particularly in the prostate, where irregular or asymmetric ablation may result in incomplete treatment or unintended injury to adjacent structures. Previous theoretical and experimental investigations have demonstrated that multi-fiber configurations improve lesion uniformity by promoting overlapping thermal fields and reducing steep temperature gradients [11-13]. The symmetric lesions observed in this study are consistent with these findings and suggest that the dual-fiber configuration facilitates uniform energy distribution within the target tissue. Predictable lesion geometry may also improve procedural planning and reproducibility in clinical settings.

TPLA has gained increasing attention as a minimally invasive treatment option for BPH. Clinical and preclinical studies have demonstrated its feasibility and safety, with reported improvements in lower urinary tract symptoms and low complication rates [7-10,15]. These findings support the growing role of focal therapies as alternatives to conventional surgical approaches. These studies have also highlighted the importance of achieving controlled and well-defined ablation zones to ensure both efficacy and safety. The well-demarcated zones of coagulative necrosis observed in the present study are consistent with these reports and support the ability of laser-based therapies to produce localized and predictable tissue effects.

In addition to immediate tissue effects, prior studies have demonstrated dynamic changes in lesion volume and tissue response following laser ablation. Experimental models have shown that ablation zones may evolve over time due to processes such as edema, necrosis, and tissue remodeling [14]. These post-ablation changes may influence long-term treatment outcomes and should be considered when evaluating therapeutic efficacy. Although the present study was limited to immediate post-ablation assessment, the reproducible lesion geometry observed provides a foundation for understanding how these lesions may evolve in vivo. These observations are consistent with prior animal studies of TPLA, which demonstrate a central zone of coagulative tissue injury surrounded by a transitional region of sublethal hyperthermia and an inflammatory response, with preservation of surrounding tissue architecture reflecting expected thermal gradients following interstitial laser energy delivery [16]. 

An important finding of this study is the absence of carbonization, vaporization, or thermal runaway across all specimens. Excessive heating leading to tissue charring or vaporization can disrupt energy transmission, reduce procedural control, and increase the risk of complications. Maintaining temperatures below critical thresholds is therefore essential for safe and effective ablation. Previous feasibility studies of TPLA have similarly reported favorable safety profiles when energy delivery is carefully controlled [8,9,15]. The findings of the present study reinforce the importance of controlled thermal dosing and suggest that the dual-fiber configuration may help maintain stable thermal conditions.

The role of fiber configuration and spacing is particularly important in determining ablation outcomes. Prior studies have emphasized that the distance between fibers directly influences thermal overlap and lesion geometry, with optimized spacing resulting in more uniform and predictable ablation zones [12,13]. In the present study, using two fibers positioned 10 mm apart resulted in consistent, symmetric lesion formation, supporting the concept that coordinated multi-fiber systems can enhance treatment precision and reproducibility.

From a clinical perspective, the ability to generate reproducible, well-contained ablation zones is highly relevant to the treatment of BPH. Focal therapies aim to relieve obstruction while minimizing damage to surrounding structures such as the urethra, neurovascular bundles, and external sphincter. The controlled thermal spread observed in this study suggests that dual-fiber laser ablation may offer advantages in achieving this balance between efficacy and safety, which remains a key objective in the evolution of minimally invasive urologic therapies.

Several limitations should be considered when interpreting these findings. This study was conducted in an ex vivo model, which does not account for perfusion-mediated heat dissipation that occurs in vivo. Tissue perfusion can significantly influence thermal distribution and may reduce the extent and geometry of ablation compared with non-perfused tissue. Additionally, the sample size was limited, and variability across a larger cohort was not assessed. The experimental design focused on immediate post-ablation effects and did not evaluate functional outcomes, histologic evolution over time, or long-term tissue remodeling. Furthermore, the bovine prostate model, while structurally relevant, may not fully replicate the anatomical and vascular characteristics of the human prostate. Statistical analysis was limited by the small sample size, and potential variability in gross lesion measurements may influence volume estimation. Moreover, the absence of physiologic perfusion and real-time imaging guidance limits direct extrapolation to clinical conditions.

Future investigations should include in vivo validation to better characterize thermal behavior in perfused tissue and to assess safety and functional outcomes. Additional studies evaluating optimization of energy delivery parameters, fiber spacing, and real-time imaging guidance will be important for improving procedural precision. Longitudinal studies assessing lesion evolution and tissue remodeling are also warranted. Ultimately, prospective clinical trials will be necessary to determine clinical efficacy, durability of symptom relief, and safety of dual-fiber laser ablation for the treatment of BPH.

## Conclusions

Dual-fiber laser ablation using the EchoLaser X4 system produces controlled, reproducible, and spatially confined thermal coagulation lesions in bovine prostate tissue under ex vivo conditions. The observed thermal behavior and consistent lesion geometry demonstrate the system’s ability to deliver predictable energy distribution within a range sufficient to induce coagulative necrosis while avoiding carbonization. The reproducibility of ellipsoidal lesion formation and limited peripheral thermal spread observed across specimens supports the precision and stability of the dual-fiber configuration for focal tissue ablation. These findings suggest that controlled multi-fiber thermal delivery may improve treatment accuracy while minimizing unintended injury to surrounding structures. In clinical settings, perfusion-related heat dissipation, including urethral cooling and blood flow within the periprostatic venous plexus, may reduce thermal accumulation and potentially result in smaller and less symmetric ablation zones compared with the stationary ex vivo bovine model. Further in vivo and clinical studies are warranted to evaluate the effects on perfusion, functional outcomes, long-term efficacy, and safety in the management of BPH.
